# Tests for candidate-gene interaction for longitudinal quantitative traits measured in a large cohort

**DOI:** 10.1186/1753-6561-3-s7-s80

**Published:** 2009-12-15

**Authors:** Dörthe Malzahn, Yesilda Balavarca, Jingky P Lozano, Heike Bickeböller

**Affiliations:** 1 Department of Genetic Epidemiology, University Medical Center, University of Goettingen, Humboldtallee 32, D-37073 Goettingen, Germany

## Abstract

For the Framingham Heart Study (FHS) and simulated FHS (FHSsim) data, we tested for gene-gene interaction in quantitative traits employing a longitudinal nonparametric association test (LNPT) and, for comparison, a survival analysis. We report results for the Offspring Cohort by LNPT analysis and on all longitudinal cohorts by survival analysis with cohort effect adjustment. We verified that type I errors were not inflated. We compared the power of both methods to detect in FHSsim data two sets of gene pairs that interact for the trait coronary artery calcification. In FHS, we tested eight gene pairs from a list of candidate genes for interaction effects on body mass index. Both methods found evidence for pairwise non-additive effects of mutations in the genes *FTO*, *PON1*, and *PFKP *on body mass index.

## Background

The Framingham Heart Study (FHS) cohorts and simulated FHS (FHSsim) data available for Genetic Analysis Workshop 16 (GAW16) provide longitudinal data on quantitative traits and include families. FHSsim data are based on the FHS individuals and family structures, replacing only the phenotypes by simulated values. For both sets of data, we compared two association approaches to test quantitative traits for gene-gene interaction. Both approaches use baseline and available follow-up measurements and require a set of independent individuals with phenotypes and genotypes. The methods are: 1) a longitudinal nonparametric association test for cohorts (LNPT) which we recently developed and 2) survival analysis with the Cox model. The LNPT is a rank-sum procedure based on the longitudinal trait measurements. Survival analysis models event times derived from the longitudinal traits as age at the first exam when the trait crossed a predefined threshold.

Both approaches remain mathematically valid regardless of trait distribution. This is most useful: quantities with mixture distributions such as the simulated trait coronary artery calcification (CAC) remain non-normal despite blom or log transformation. Also, LNPT and survival analysis are invariant with respect to monotone transformations such as blom or log: they change neither the order (ranks) of the trait data nor the event times, given that the threshold is also transformed.

We analyzed quantitative traits with a skewed distribution: CAC in the FHSsim data and body mass index (BMI) in the FHS data. We verified that the type I error of 5% was kept, applying our methods on the original, untransformed data. CAC is influenced by two separate sets of interacting gene pairs according to the GAW16 answers [1]. We computed the power of the two approaches to detect two-locus interactions for the two interacting gene pairs at the α = 5% level in all 200 data replicates. In FHS, we analyzed BMI for gene-gene interactions based on a set of candidate genes that carry susceptibility variants that have been identified in previous single-locus studies. We report results for the offspring cohort by LNPT analysis, and on all longitudinal cohorts by survival analysis with cohort effect adjustment. GAW16 data retrieval and all analyses were carried out in compliance with the Helsinki Declaration.

## Materials

The FHS data provide 227 independent individuals and 980 pedigrees. For association analysis, 1631 (233 Original (OC), 1243 Offspring (Off), and 155 Third Generation (Gen3) Cohort) unrelated individuals had available genotype and phenotype data that could be extracted, including founders and one random individual from all remaining multimember pedigrees. Similar selection procedures were applied to FHSsim.

For FHS, Gen3 provides baseline values only. OC and Off provide four consecutive measurements per individual. The LNPT requires that individuals are followed up at the same intervals but tolerates missing trait values. We harmonized follow-up times by allocating five examination time points T = 1, ..., 5 (baseline, 6 yr, 12 yr, 19 yr, 26 yr after baseline). T = 2 is missing for Off, T = 5 is missing for OC. Longitudinal analysis by LNPT used the phenotypes from all time points when both cohorts were examined (T = 1, 3, 4). Some individuals have incomplete longitudinal phenotypes. For FHSsim, each cohort (Gen3, OC, Off) provides simulated baseline trait values and two follow-ups, each 10 years apart. All longitudinal phenotypes are complete.

For survival analysis, time to event was defined as the earliest age at which the individual presented a BMI ≥ 25 kg/m^2 ^(FHS) or a value of CAC>230 (FHSsim, no unit). For censored individuals, time to censoring was age at the last available exam. Censoring occurred when individuals did not pass the threshold either for any of the longitudinal measurements or for an initial sequence of longitudinal measurements after which they are missing to follow up. We excluded individuals who have baseline trait values which are either missing or above the threshold.

For analysis of CAC (FHSsim), we selected the two interacting SNP pairs from the GAW answers [1] (single-nucleotide polymorphisms (SNPs) τ1 and τ2; τ3 and τ4). τ1 displays only a minimal main effect, τ2 a measurable additive main effect. τ3 and τ4 are epistatic SNPs. All four SNPs have minor allele frequencies (MAF) of approximately 0.5.

For gene-gene interaction analysis of BMI (FHS), we selected five candidate SNPs previously found to be associated with alteration of BMI [2]. The SNPs are rs6602024 (MAF 11%, chromosome 10, gene *PFKP*); rs1121980 and rs9930506 (both: MAF 44%, chromosome 16, gene *FTO*); and rs854560 (MAF 37%, chromosome 7, gene *PON1*) and rs6971091 (MAF 22%, chromosome 7, gene *FAM137A*).

## Methods

### LNPT analysis

The LNPT tests for association of longitudinal quantitative traits with respect to a set of influencing factors, which divide the cohort into subgroups. The LNPT tests the null hypothesis of no difference in trait distribution **F **between these subgroups

where **C **is a contrast matrix and **F **= {} is the set of distribution functions  ordered by observational time point *t *and the influencing factors (*kls*) of interest. For gene-gene interaction analysis, two factors *k*, *l *are used to code for SNP genotype at the two loci (*k*, *l *= 0,1,2 for biallelic loci) and we stratified by sex *s *= {*m*, *w*}. The test statistic resembles structurally a heteroscedastic repeated measures ANOVA which is performed on the mid-ranks of the longitudinal traits [3], estimating longitudinal covariance from the ranks without assuming any structure. Trait vectors **Y**_*i *_can be arbitrarily dependent between exams/time points *t *of the same individual *i *while they are assumed to be independent between individuals. Individuals should be followed up at the same time intervals. The LNPT yields a set of adjusted *p*-values for tests of average effects of the loci, sex, and number of exam and for tests of all interactions. The LNPT can handle missing values for the longitudinal phenotype. The test statistic is computed using the existing phenotype measurements when values are missing. The LNPT can model covariates as additional factors. For all tested interactions, each stratum must contain a minimum of ten observations per exam to ensure consistent estimates of the test statistic.

Prior to genetic analysis, we tested for a cohort effect (FHS: OC, Off; FHSsim: OC, Off, Gen3) by using factors for cohort type and for sex, omitting the two genetic factors *k*, *l*. Gene-gene interaction analysis was restricted to the large Offspring sample (*N *= 1243), to individuals with no cholesterol treatment (79% of Off sample). For BMI (FHS), we restricted baseline age to the interval 25-46 yr to avoid effects of BMI-related mortality (manifest as stagnating median BMI for old age). We adjusted for age by using a two-level factor, testing only age-averaged interaction effects. Levels are assigned according to baseline age with median baseline age as break point.

### Survival analysis: Cox proportional hazards model and time-varying covariates

Survival analysis evaluates the time to the occurrence of an event of interest and its dependence on particular characteristics such as sex, genotype, and cohort. We employ the Cox proportional hazard model and its extension for time-varying factors [4], which are smoking status and cholesterol treatment. The extended Cox model compares the risk of an event between two or more subgroups at each event time, where the risk group of an individual changes according to the time-varying factor. The model was adjusted for a cohort effect using all cohorts with follow-up measurements (FHS: OC, Off; FHSsim: OC, Off, Gen3). The likelihood-ratio test was used to test for the gene-gene interaction by comparing the log-likelihood of the model with interaction against the null hypothesis model of no interaction.

## Results

A significant main effect of cohort type and a significant interaction of cohort type with sex were detected in FHS BMI data (LNPT) and FHSsim CAC data (LNPT and Cox model). We report gene-gene interaction analysis on the Offspring Cohort (LNPT) and on all longitudinal cohorts (Cox model with cohort effect adjustment).

Rates of false positives (i.e., type I errors) for *p*-values ≤ 0.05 were estimated for the interaction tests for SNP pairs rs854560 and rs1121980 (FHS) and τ1, τ2 (FHSsim) after permutation of the assignment between longitudinal phenotypes and individuals. Permutation destroys associations but retains the distributional properties of the trait.

### FHSsim: results for CAC

The tested SNP interactions τ_1_, τ_2 _and τ_3_, τ_4 _are known to contribute to the analyzed trait CAC [1]. For each SNP pair, power is estimated as percentage of significant results (*p *≤ 0.05) of the interaction test on the 200 replicates. The LNPT detected both interactions with 100% power in the largest ascertainable sample (*n *= 856 on average) and with 99% (τ_1_, τ_2_) and 100% (τ_3_, τ_4_) for sample size *n *= 400. The Cox model had a power of 94% (τ_1_, τ_2_) and 100% (τ_3_, τ_4_) for the largest ascertainable sample (*n *= 808 on average, 49% events) but only 76% (τ_1_, τ_2_) and 97% (τ_3_, τ_4_) for sample size *n *= 400. The Cox model did benefit from cohort effect adjustment: SNP interaction τ_1_, τ_2 _was found with 94% power after adjustment instead of 69% with no adjustment (*n *= 808). For CAC, the LNPT is more powerful compared with survival analysis. Estimates of type I error at the α = 5% level were 4.76% (LNPT) and 4.90% (Cox).

### FHS: results for BMI

A significant cohort effect was detected by LNPT (*p*_cohort _= 0.0001, *p*_cohort × sex _= 0.03). It is due to men (LNPT, stratified analysis). The Cox model yields threshold-dependent results (event BMI>25: *p*_cohort _= 0.15, *p*_cohort × sex _= 0.13, event BMI>30: *p*_cohort _= 0.02, *p*_cohort × sex _= 0.80) detecting no interaction between cohort and sex. In the remainder of this paper, we report results on the Cox model with threshold BMI>25 (yielding more events) and cohort-effect adjustment.

The two approaches yield consistent results with evidence for pairwise interaction between the three genes *FTO *(rs1121980 and rs9930506), *PON1 *(rs854560), and *PFKP *(rs6602024). *p*-Values for gene-gene interaction on BMI are given in Table 1, highlighting significant values (*p *≤ 5%). For SNPs rs6602024 and rs6971091, LNPT analysis distinguished only between carriers and non-carriers of the rare minor allele. For SNP rs6602024, minor allele homozygotes were excluded from survival analysis. Survival analysis computes a single interaction *p*-value for each SNP pair by the likelihood-ratio test comparing the interaction model against the null model of no gene-gene interaction. In contrast, the LNPT yields a set of *p*-values for the interaction tests that are adjusted to the α = 5%-level. In the present data, gene-gene interaction is detected as effect on time-course (see Table 1). Estimates of type I error at the α = 5% level were 4.99% (LNPT, with and without age adjustment) and 4.80% (Cox). To quantify effect size, we report the hazard ratios (HR) obtained by survival analysis. The findings for main effects agree with previous reports: rs854560 genotype AA yields higher BMI (AA vs. TT, HR = 1.76 (95%CI: 1.21-2.51); AA vs. AT, HR = 1.24, (95%CI: 0.97-1.50)). The rare rs6602024 genotype AG was previously reported to yield higher BMI (AG vs. GG, HR = 1.16 for our sample (95%CI: 0.89-1.54)). For the combination, highest BMIs are obtained when rs854560 genotype TT is combined with rs6602024 genotype GG (TT*GG vs. AA*AG, HR = 3.7 (95%CI: 0.82-16.42, small sample size problem with rare two-locus genotype groups)). The combined effect of rs854560 with rs9930506 shows the highest BMIs when rs854560 genotype AT is combined with genotype GA or GG of marker rs9930506 (AT*GA vs. AA*AA, HR = 2.3 (95%CI: 1.40-3.84). SNP rs6602024 with rs1121980 shows the highest BMI values when rs6602024 genotype AG is combined with genotype GA or GG of marker rs1121980 (AG*GA vs. GG*AA yields HR = 2.7 (95%CI: 1.26-5.84); AG*GG vs. GG*AA yields HR = 1.78 (95%CI: 0.77-4.16)).

**Table 1:** Gene-gene interaction for BMI in FHS data^a^

	LNPT analysis of independent individuals in Offspring Cohort^b ^(*n *= 824) for the trait Longitudinal BMI		Survival analysis of independent individuals in Original and Offspring Cohorts (*n *= 858) for the trait Event Overweight (BMI ≥ 25)	
	
Modelled factors	Sex and two gene factors		Sex, cohort, and two gene factors	
		
Age adjustment	No	Yes	Age is event time	
		
Time-varying covariates	No^b^	No^b^	No	Yes^h^
SNP pair				

rs854560 and rs6602024^c-*e*^				
analyzing exams (T = 1, 4)^d^	**0.022^g^**	**0.040^g^**	**0.003**	**0.007**
analyzing exams (T = 1,3,4)^d^	0.092^g^	n.s.	**0.003**	**0.007**
rs854560 and rs1121980	**0.034^f^**	**0.003^f^**	**0.030**	0.053
rs854560 and rs9930506	**0.048^f^**	**0.004^f^**	**0.005**	**0.008**
rs6971091^c ^and rs6602024^c, *e*^	n.s.	n.s.	n.s.	n.s.
rs6971091^c ^and rs1121980	n.s.	n.s.	n.s.	n.s.
rs6971091^c ^and rs9930506	n.s.	n.s.	n.s.	n.s.
rs6602024^c, *e *^and rs1121980	0.053^g^	**0.049^g^**	**0.020**	**0.028**
rs6602024^c, *e *^and rs9930506	n.s.	n.s.	0.077	n.s.

^a^*p*-Values ≤ 0.1 are given, otherwise marked as not significant (n.s.). Bold font indicates *p*-values ≤ 0.05.

^b^Individuals with no cholesterol treatment and baseline age 25-46 yr, Exams T = 1,3,4.

^c^Carriers of the rare allele for the SNP were pooled for LNPT analysis.

^d^SNP pair has a small two-locus genotype strata. LNPT analysis was performed for two sets of exams. Survival analysis used all available event times.

^e^Minor allele homozygotes for this SNP were omitted for survival analysis.

^f^Interaction of SNP1-SNP2 with number of exam (averaged for sex).

^g^Interaction of SNP1-SNP2 with sex and exam number.

^h^Inclusion of time-varying covariates smoking status and cholesterol treatment.

## Discussion

Transformation from follow-up data to event-time data can be viewed as reduction of information content. It requires a priori a well reasoned threshold value. Results of survival analysis depend on the choice of this threshold. We illustrated this for the detection of the cohort effect (BMI, FHS). For some scenarios, it may well be possible that longitudinal data can not be analyzed by survival analysis. For example, if a particular two-locus genotype would efficiently protect against overweight, one could face the problem that the threshold either provides insufficient contrast or does not yield sufficient numbers of events for analysis for the two-locus genotypes of interest. For BMI, an established threshold value of 25 kg/m^2 ^for the status overweight was available. In contrast to CAC, distribution of BMI is almost normal (only slightly skewed) and candidate SNPs for BMI tend to be rare, yielding small two-locus genotype subgroups. For the SNP-pair with smallest subgroup size (rs854560 and rs6602024), the likelihood-ratio test performed for survival analysis was observed to yield significance although simultaneously, the computed confidence intervals for the HR ratios were not significant but included very high HRs.

Survival analysis has the benefit that it yields hazard ratios as readily interpretable estimates of effect size. The LNPT tests for a much more general feature, namely differences between whole trait distributions without making assumptions about an underlying model. Consequently, it primarily provides a tool for testing for group differences but does not offer readily interpretable estimates of effect size. When a set of factors are chosen, by default the LNPT also tests their interactions, with the consequence that interactions with weak marginal effects are less likely to be missed.

## Conclusion

CAC has a strongly skewed distribution. For parametric approaches, this would cause loss of power or inflated rates of false positives. Neither is the case for the LNPT and survival analysis. We have shown that they both keep the rate of false positives at the 5% level while they successfully detected gene-gene interactions for simulated and real phenotypes with good power. The LNPT was found to be more powerful compared with survival analysis for detection of a cohort effect in BMI (FHS) and more powerful for detection of gene-gene interaction, particularly for CAC (FHSsim). The LNPT is a longitudinal approach. In its ability to incorporate loss to follow up, the LNPT is better than survival analysis: the LNPT uses all remaining trait measurements, whereas for survival analysis these individuals are likely to become censored or even must be excluded in case of missing baseline value.

For high-dimensional data such as genome-wide studies, sophisticated boosting techniques exist for survival analysis [5]. They yield sparse models with high explanatory power. For the LNPT, data partition techniques similar to tree approaches or multifactor dimensionality reduction could be used. Further research is needed on this issue.

## List of abbreviations used

BMI: Body mass index; CAC: Coronary artery calcification; FHS: Framingham Heart Study; FHSsim: Framingham Heart Study simulated data; GAW16: Genetic Analysis Workshop 16; Gen3: Third Generation Cohort; HR: Hazard ratio; LNPT: Longitudinal nonparametric association test; MAF: Minor-allele frequency; OC: Original Cohort; Off: Offspring Cohort; SNP: Single-nucleotide polymorphism

## Competing interests

The authors declare that they have no competing interests.

## Authors' contributions

DM designed and coordinated the study, carried out statistical analyses, and drafted the manuscript. YB carried out statistical analyses. JPL participated in data preparation. DM and HB conceived the study. All authors contributed to the writing of the manuscript. All authors read and approved the final manuscript.
